# Defining the Akt1 interactome data and delineating alterations in its composition as a function of cell cycle progression

**DOI:** 10.1016/j.dib.2017.02.018

**Published:** 2017-02-12

**Authors:** Shweta Duggal, Noor Jailkhani, Mukul Kumar Midha, Kanury V.S. Rao, Ajay Kumar

**Affiliations:** a International Centre for Genetic Engineering and Biotechnology (ICGEB), Aruna Asif Ali Marg, New Delhi 110067, India; b Drug Discovery Research Center (DDRC), Translational Health Science and Technology Institute (THSTI), NCR Biotech Science Cluster, 3rd Milestone, Faridabad-Gurgaon Expressway, Faridabad 121001, Haryana, India

**Keywords:** Akt1, Cell cycle, Interactome, SILAC, AP-MS

## Abstract

Akt1 is a multi-functional protein implicated in key cellular processes including regulation of proliferation, survival, metabolism and protein synthesis. Its functional diversity results through interactions with other proteins which change with changing context. This study was designed to capture proteins, which interact with Akt1 as the cell cycle progresses from G0 to G1S and then G2 phase. Such an insight might help us understand the role of Akt1 in cell cycle, which as of now is not well explored. Akt1 expressing HEK 293 cells were cultured in light, medium and heavy labeled SILAC media. Normal lysine and arginine were incorporated as light labels; 6 Da (Dalton) heavier isotopes of the same amino acids were used as medium labels; while for heavy labeling the isotopes were 8 and 10 Da heavier. Light labeled cells were arrested in G0 phase while medium and heavy labeled cells were arrested in G2 and G1S phases, respectively. Equal number of cells from each phase was pooled, lysed and subjected to Affinity Purification coupled to Mass Spectroscopy (AP-MS). The obtained Akt1 protein partners were observed to change as the cell cycle progressed from G0 to G1S and then to G2 phase. Additionally, SILAC labeling aided in quantitative estimation of changing association of a number of proteins which were common to two or more phases, with Akt1. Data are available via ProteomeXchange with identifier PXD005557.

**Specifications Table**

**Table:** 

Subject area	*Biology*
More specific subject area	*Proteomics*
Type of data	*Mass Spectrometry raw files, Figure*
How data was acquired	*Mass Spectroscopy, AB Sciex 5600 Triple TOF*
Data format	*Raw and analyzed*
Experimental factors	*Stable Akt1 cell line arrested at different cell cycle stages: G0, G1S and G2 phase.*
Experimental features	*SILAC labeling, LysC - Trypsin Digestion, peptide separation through nano-LC and MS/MS analysis on AB SCIEX 5600 triple-TOF mass spectrometer*
Data source location	*New Delhi, India*
Data accessibility	*Data is within this article and available in a Public repository via ProteomeXchange with identifier ProteomeXchange:*PXD005557

**Value of the data**

• Our data highlights changing Akt1 interacting protein partners as the cell cycle progresses from G0 to G1S and then G2 in HEK 293 cell line.
• A number of proteins common to 2 or more cell cycle phases present altered association with Akt1 protein as the cell cycle progresses.
• This data might help in understanding how Akt1 extends its regulatory effect upon cell cycle progression.
• Proteins showing altered association with Akt1, as the cell cycle progresses, can be further explored as targets in regulating cell division.

## 1 Data

**Fig. 1:**
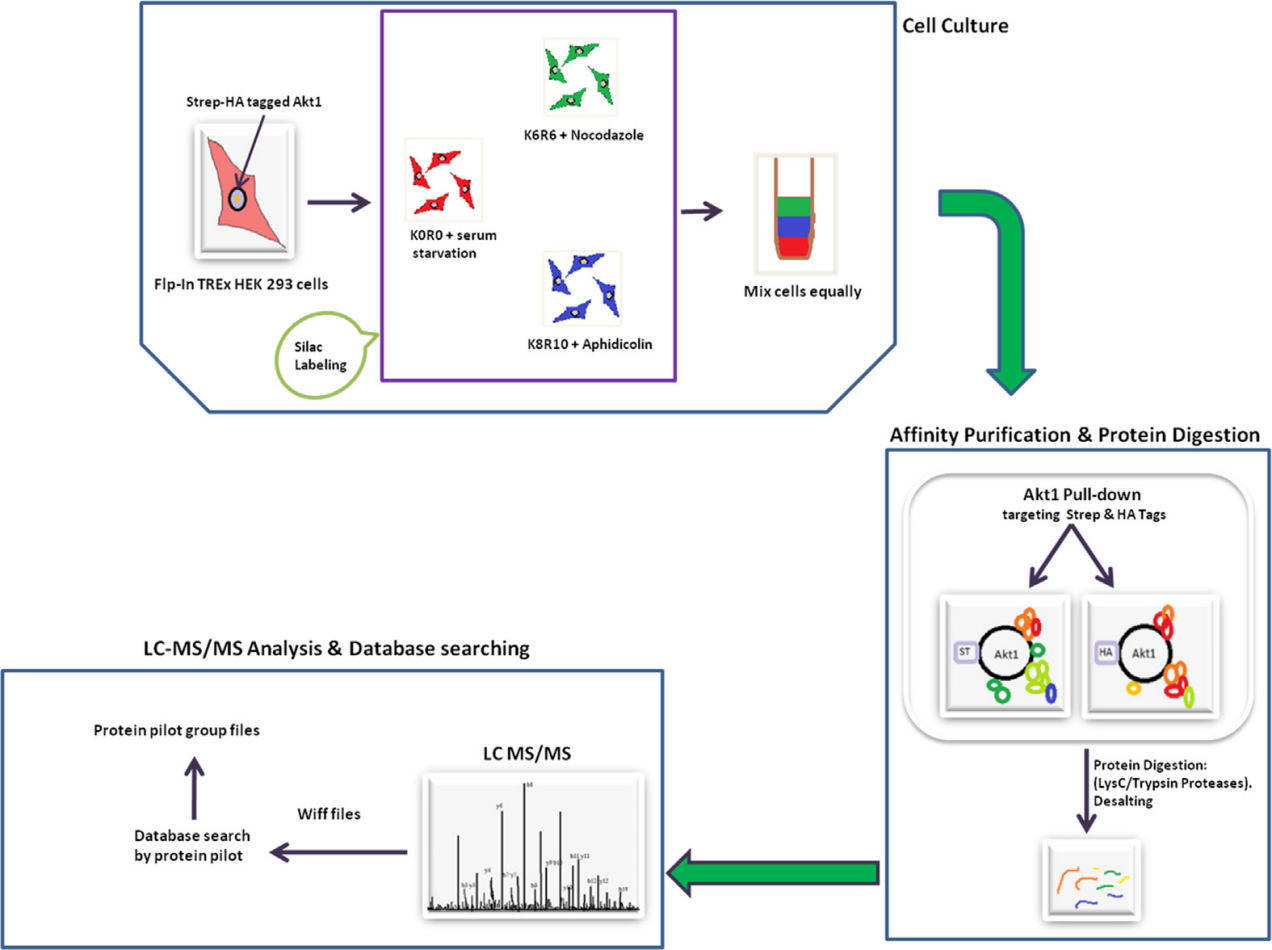
Schematic overview showing the experimental workflow.

Akt1 expressing HEK 293 cells were SILAC labeled to capture dynamic changes in Akt1 interactome as the cell cycle progresses from G0 to G1S and then to G2 phase. Three biological replicates were processed and analyzed. Affinity purified samples were double digested followed by acquisition and the results were obtained as 6 RAW file pairs (wiff and corresponding wiff.scan files) from AB SCIEX 5600 triple TOF instrument. Wiff files were submitted to Protein Pilot software version 4.5 and resulted in 18 protein pilot group files (6 group files per biological replicate). The experimental overview is as shown in Fig. 1. Data is publicly available via ProteomeXchange with identifier ProteomeXchange: PXD005557.

## 2 Experimental design, materials and methods

### 2.1 Generation of stable cell line

To generate tetracycline (Tet) inducible Akt1 expression vector, pENTR221 entry vector with Akt1 ORF (GE Dharmacon) was selected for LR recombination along with modified destination vector (pcDNA/FRT/TO); latter was a kind gift from Dr. Matthias Gstaiger (Institute of Molecular Systems Biology, ETH Zurich, Zurich, Switzerland). Destination vector was modified to include a Strep-HA tag for selective separation of Akt1 protein.

Flp-In T-REx HEK 293 cells (Invitrogen), stably expressing the Tet-repressor, were cultured in 10% DMEM medium containing 100 µg/ml zeocin and 15 µg/ml blasticidin. Akt1 expression vector was co-transfected with pOG44 vector in these cells using Xtremegene 9 transfection reagent (Roche), as per manufacturer׳s instructions.

Two days after transfection, cells were transferred to hygromycin selection media (@ 75 µg/ml) with 10% Tet screened FBS. Selection was continued over 2 weeks with media change twice a week. Optimal Tet dosage, required to induce Akt1 expression, was determined by adding Tet to culture media at Tet concentrations ranging from 0.1 µg/ml to 5 µg/ml. Stable Akt1 expression was obtained at 1 µg/ml Tet concentration. Tet (@ 1 µg/ml) was supplemented in media of Akt1 expressing cells every 24 h to maintain Akt1 expression. Akt1 expression was validated using anti-HA antibody (#SC-7392; mouse monoclonal, dilution 1:3000, from Santa Cruz) by western blotting. Endogenous Akt1 levels were also checked with anti-Akt1 antibody (C73H10; rabbit monoclonal, dilution 1:1000; Cell Signaling).

### 2.2 SILAC (Stable Isotope Labeling in Cell Culture) labeling

Akt1 expressing HEK-293 cells were expanded and cultured in SILAC media containing “light” (K0R0), “medium” (K6R6) or “heavy” (K8R10) isotopes of lysine and arginine for at least 5 cell doublings to allow complete label incorporation [1]. At 70% confluency, Tet was supplemented to culture and 24 h later K0R0 labeled cells were arrested in G0 phase by overnight serum starvation [2]; K8R10 labeled cells were arrested in G1S phase following 16–18 h incubation in presence of 5 µg/ml aphidicolin [3]; and K6R6 labeled cells were arrested in G2 phase following 16–18 h incubation in presence of 400 ng/µl Nocodazole [4]. The arrested cells were trypsinized; counted by trypan blue staining method; and pelleted separately by centrifugation at 1500 rpm for 10 min. Multiple cell pellets were generated for each cell cycle phase and stored at −80 °C, until used.

### 2.3 Streptactin-HA (SH) tagged Affinity Purification

Same number of Akt1 expressing cells arrested in G0, G1S and G2 phases were pooled and lysed for 1 h on ice in IP buffer (150 mM NaCl; 50 mM Tris–HCl pH7.5; 1% NP-40; 1× protease inhibitor cocktail and 1 mM PMSF) [5]. Cell lysates were cleared off debris following centrifugal separation at 10,000 rpm for 15 min at 4 °C. Akt1 protein complexes were selectively purified in parallel sets by targeting Strep and HA tags simultaneously as instructed in kit manuals (Invitrogen). Briefly, Strep-tagged Akt1 protein complexes were mixed with 100 µl of Streptactin magnetic beads and kept for 1-h incubation on a rotary shaker at 4 °C. Any non-specific interactors were washed away after five consecutive washes with five bead volumes of streptactin wash buffer. Akt1 protein and its interactors were finally eluted in two elution steps; with 125 µl Strep Elution buffer per elution step. For purification of HA-tagged Akt1 protein complexes, cleared cell lysates were kept for incubation with 50 µl of pre-washed HA agarose beads for 2 h at 4 °C on a rotary shaker. After 3 quick washes with ten bead volumes TBS-T buffer, Akt1 protein complexes were eluted thrice; with 50 µl HA peptide (250 µg/ml) per elution step. Akt1 protein partners were purified as mentioned above for three biological replicates and the eluates (Strep and HA eluates) for each replicate set were pooled, lyophilized and saved at −80 °C, until further processing.

### 2.4 Protein digestion and sample preparation for LC–MS/MS

Each biological replicate was processed for protein digestion separately. Lyophilized eluates were re-suspended in 40 µl of 100 mM ammonium bicarbonate, vortexed well and supplemented with 2 µl of 2% SDS (denaturant buffer). Protein samples were reduced at 60 °C with 4 µl of 50 mM TCEP [Tris-(2-carboxyethyl) phosphine] for 1 h. Further, reduced cysteine residues were blocked with 2 µl of 200 mM MMTS (methyl methanethiosulfonate) at room temperature during 10 min incubation. Digestion was initiated by adding 5 µl of 0.1 µg/µl endo-proteinase LysC and kept for incubation for 4 h in a 37 °C water bath. After a short spin, 1 µl of trypsin (1 µg/µl) was supplemented and continued incubation for another 12–16 h at 37 °C. A drop of formic acid was used to terminate protein digestion.

Acidified samples were lyophilized followed by peptide purification using monospin C-18 columns (Waters). Pre-conditioned C-18 columns were equilibrated with 3% acetonitrile (ACN) in 0.1% formic acid (FA). The lyophilized peptide samples were dissolved in 3% ACN in 0.1% FA and loaded on to C-18 columns and allowed to bind for 10 min. The samples were passed twice through the columns to ensure complete binding. After 10 stringent washes with 3% ACN in 0.1% FA, the digested peptides were eluted first in 40% ACN followed by two elutions in 60% ACN. Finally, the three eluates were pooled and lyophilized.

In an effort to remove any remaining salt, the eluted peptides were re-dissolved in 500 µl of 5 mM ammonium formate (pH 2.5) in 30% ACN and gently vortexed to mix. The cation exchange cartridge was fixed and conditioned before loading the sample. Following sample loading, the cartridge was washed thrice with 1 ml of 5 mM ammonium formate. The peptides were re-eluted twice with 400 µl of 500 mM ammonium formate (pH 2.5) in 30% ACN, pooled and lyophilized.

### 2.5 LC–MS/MS analysis

All samples were analyzed by nano-flow liquid chromatography (LC) on a nanoflex system (Eksigent Technologies, AB SCIEX) coupled to a triple TOF 5600 Mass Spectrometer (AB SCIEX; Concord, Canada). Each biological replicate was injected twice as two technical replicates; LC solvents included mobile phase A (2% ACN in 0.1% FA) and mobile phase B (98% ACN in 0.1% FA). For optimal sample delivery reproducibility, the auto-sampler was operated in full injection mode overfilling the 1 µl loop with 3 µl sample. Peptides were first run through trap column for 30 min and then eluted out from analytical column (chromolith column-particle size 5 µm, length 15 cm, 75 µm ID) with a flow rate of 300 nl/min. A gradient of solvent B from 5% to 60% was followed for 80 min while that of 60–90% for 2 min. The column was regenerated by washing with 90% solvent B for 6 min and re-equilibrated with 5% solvent B for 22 min. Auto-calibration was done using 25 fmol of beta-galactosidase after every two runs. Peptides were injected into mass spectrometer by using 10 μm SilicaTip electrospray PicoTip emitter and the eluted peptides were monitored by following ion source parameters – IHT = 130°, ISVF = 2.1 kv, GS1 = 20, curtain gas = 25. MS data was acquired in information-dependent acquisition (IDA) mode using Analyst TF 1.6 software. Mass spectra and tandem mass spectra were recorded in positive-ion and “high-sensitivity” mode; LC–MS/MS analysis was performed using TOF-MS survey scan from 350 to 1250 *m/z*. Accumulation time of scan was 500 ms, followed by fragmentation of 10 most abundant ion peaks. Accumulation time was set to 200 ms. Rolling collision energy was automatically controlled by IDA rolling collision energy parameter script. Selection criteria for parent ion to be fragmented included intensity – where ions had to be greater than 150 cps, mass tolerance of 50 mDa, with a charge state of +2 to +5. The ions, once fragmented, were excluded from further fragmentation for 12 s.

### 2.6 Database search

A set of six mass spectrometry wiff files were generated for each biological replicate. The two technical replicate wiff files were pooled-in before searching against uniprot_human swissprot database (Release April 2015) using protein pilot version 4.5 (revision no. 1656). Following settings were used for paragon searches - Species as Homo sapiens; LysC and Trypsin as enzyme categories for different runs; 2 missed cleavages allowed; with cys alkylation as Methyl methanethiosulphonate (MMTS). Identification, SILAC (Lys+6, Arg+6) and SILAC (Lys+8, Arg+10) as sample types and the “Search Effort” parameter “Thorough ID”, which gives us a broad search of various protein modifications. The following parameters were used for identification and quantification of differentially expressed proteins - (1) Auto Bias correction for heavy to light ratio. (2) Threshold of 1% accepted Global False discovery rate from fit (G-FDR-fit) proteins; (3) Minimum protein confidence threshold cutoff of 95%; (4) At least one peptide with 95% confidence for the relative expression.

For each biological replicate, six protein pilot files were generated: files representing 3 SILAC labels (light, medium and heavy) with both the enzymes used for digestion (LysC and Trypsin) were created individually. Here, light was represented as 0/0 (G0 stage), medium as 6/6 (G2 stage) and heavy as 8/10 (G1S stage).

The mass spectrometry proteomics data have been deposited to the ProteomeXchange Consortium via the PRIDE [6] partner repository with the dataset identifier PXD005557.

## Figures and Tables

**Figure:**
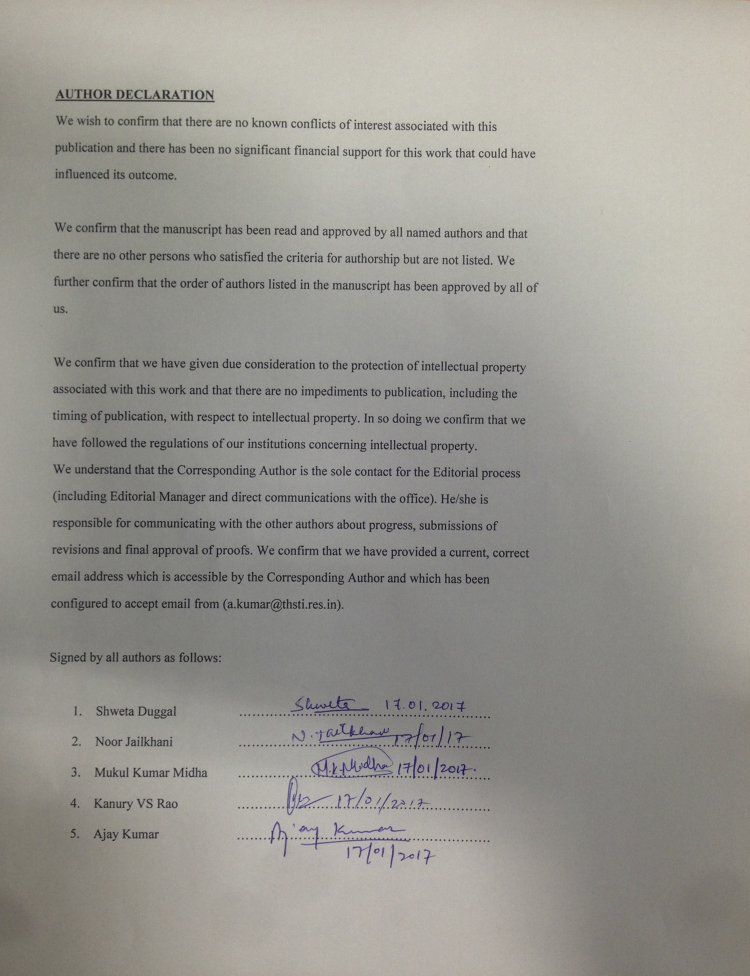
Supplementary Fig.
